# Supplementary material for: Role and mechanism of NCAPD3 in promoting malignant behaviors in gastric cancer
Source: Front Pharmacol. 2024 Apr 22;15:1341039. doi: 10.3389/fphar.2024.1341039 (PMC11070777; doi:10.3389/fphar.2024.1341039)
Supplement: Supplementary file 11 [file DataSheet2.ZIP › GSEA/Oncogenic signature/my_analysis.Gsea.1599461962066/STK33_DN.html]

Details for gene set STK33\_DN[GSEA]

|  || Dataset | filtered\_dataset.sample\_info.cls#WT\_versus\_NCAPD3\_MUT |
| Phenotype | sample\_info.cls#WT\_versus\_NCAPD3\_MUT |
| Upregulated in class | WT |
| GeneSet | STK33\_DN |
| Enrichment Score (ES) | 0.2164956 |
| Normalized Enrichment Score (NES) | 1.1135638 |
| Nominal p-value | 0.31674957 |
| FDR q-value | 0.70262975 |
| FWER p-Value | 1.0 |
Table: GSEA Results Summary

  

Fig 1: Enrichment plot: STK33\_DN      
 Profile of the Running ES Score & Positions of GeneSet Members on the Rank Ordered List

  

| SYMBOL | TITLE | RANK IN GENE LIST | RANK METRIC SCORE | RUNNING ES | CORE ENRICHMENT || 1 | 388969 | C2orf68 | 10 | 1.097 | 0.0462 | Yes |
| 2 | 84133 | ZNRF3 | 30 | 0.996 | 0.0809 | Yes |
| 3 | 323 | APBB2 | 35 | 0.977 | 0.1255 | Yes |
| 4 | 153364 | MBLAC2 | 79 | 0.838 | 0.1351 | Yes |
| 5 | 80759 | KHDC1 | 85 | 0.818 | 0.1713 | Yes |
| 6 | 51540 | SCLY | 95 | 0.800 | 0.2037 | Yes |
| 7 | 79109 | MAPKAP1 | 130 | 0.770 | 0.2165 | Yes |
| 8 | 51018 | RRP15 | 192 | 0.685 | 0.2056 | No |
| 9 | 403313 | PLPP6 | 274 | 0.615 | 0.1767 | No |
| 10 | 6419 | SETMAR | 296 | 0.593 | 0.1903 | No |
| 11 | 57460 | PPM1H | 502 | 0.472 | 0.0644 | No |
| 12 | 4548 | MTR | 592 | 0.428 | 0.0207 | No |
| 13 | 55612 | FERMT1 | 662 | 0.396 | -0.0102 | No |
| 14 | 134430 | WDR36 | 733 | 0.359 | -0.0435 | No |
| 15 | 84451 | KIAA1804 | 751 | 0.348 | -0.0390 | No |
| 16 | 25825 | BACE2 | 756 | 0.344 | -0.0251 | No |
| 17 | 256435 | ST6GALNAC3 | 766 | 0.341 | -0.0151 | No |
| 18 | 5723 | PSPH | 859 | -0.267 | -0.0688 | No |
| 19 | 4680 | CEACAM6 | 955 | -0.374 | -0.1196 | No |
| 20 | 978 | CDA | 1023 | -0.421 | -0.1478 | No |
| 21 | 222166 | MTURN | 1030 | -0.429 | -0.1313 | No |
| 22 | 6590 | SLPI | 1062 | -0.453 | -0.1317 | No |
| 23 | 84706 | GPT2 | 1076 | -0.460 | -0.1188 | No |
| 24 | 2706 | GJB2 | 1115 | -0.490 | -0.1225 | No |
| 25 | 352909 | DNAAF3 | 1152 | -0.515 | -0.1236 | No |
| 26 | 27074 | LAMP3 | 1158 | -0.522 | -0.1018 | No |
| 27 | 831 | CAST | 1160 | -0.524 | -0.0770 | No |
| 28 | 440 | ASNS | 1232 | -0.609 | -0.0989 | No |
| 29 | 26999 | CYFIP2 | 1234 | -0.611 | -0.0699 | No |
| 30 | 202 | AIM1 | 1290 | -0.681 | -0.0767 | No |
| 31 | 8501 | SLC43A1 | 1296 | -0.690 | -0.0467 | No |
| 32 | 7414 | VCL | 1308 | -0.712 | -0.0201 | No |
| 33 | 54463 | FAM134B | 1356 | -0.815 | -0.0145 | No |
| 34 | 5055 | SERPINB2 | 1397 | -1.088 | 0.0094 | No |
Table: GSEA details [plain text format]

  

Fig 2: STK33\_DN      
 Blue-Pink O' Gram in the Space of the Analyzed GeneSet

  

Fig 3: STK33\_DN: Random ES distribution      
 Gene set null distribution of ES for **STK33\_DN**
